# Associations between abdominal adipose tissue, reproductive span, and brain characteristics in post-menopausal women

**DOI:** 10.1016/j.nicl.2022.103239

**Published:** 2022-10-26

**Authors:** Louise S. Schindler, Sivaniya Subramaniapillai, Claudia Barth, Dennis van der Meer, Mads L. Pedersen, Tobias Kaufmann, Ivan I. Maximov, Jennifer Linge, Olof Dahlqvist Leinhard, Dani Beck, Tiril P. Gurholt, Irene Voldsbekk, Sana Suri, Klaus P. Ebmeier, Bogdan Draganski, Ole A. Andreassen, Lars T. Westlye, Ann-Marie G. de Lange

**Affiliations:** a LREN, Centre for Research in Neurosciences, Department of Clinical Neurosciences, Lausanne University Hospital (CHUV) and University of Lausanne, Lausanne, Switzerland; b Department of Psychology, University of Oslo, Oslo, Norway; c NORMENT, Division of Mental Health and Addiction, Oslo University Hospital & Institute of Clinical Medicine, University of Oslo, Oslo, Norway; d Department of Psychiatric Research, Diakonhjemmet Hospital, Oslo, Norway; e School of Mental Health and Neuroscience, Faculty of Health Medicine and Life Sciences, Maastricht University, The Netherlands; f Department of Psychiatry and Psychotherapy, University of Tübingen, Germany; g Department of Health and Functioning, Western Norway University of Applied Sciences, Bergen, Norway; h AMRA Medical AB, Linköping, Sweden; i Department of Health, Medicine, and Caring Sciences, Linköping University, Linköping, Sweden; j Center for Medical Image Science and Visualization (CMIV), Linköping University, Linköping, Sweden; k Department of Psychiatry, University of Oxford, Oxford, UK; l Wellcome Centre for Integrative Neuroimaging, University of Oxford, Oxford, UK; m Dept. of Neurology, Max Planck Institute for Human Cognitive and Brain Sciences, Leipzig, Germany; n KG Jebsen Centre for Neurodevelopmental Disorders, University of Oslo, Oslo, Norway

**Keywords:** Brain age, White matter hyperintensities, Adipose tissue, Cardiometabolic health, Body MRI, Menopause, Reproductive span, Polygenic scores, UK Biobank

## Abstract

The menopause transition involves changes in oestrogens and adipose tissue distribution, which may influence female brain health post-menopause. Although increased central fat accumulation is linked to risk of cardiometabolic diseases, adipose tissue also serves as the primary biosynthesis site of oestrogens post-menopause. It is unclear whether different types of adipose tissue play diverging roles in female brain health post-menopause, and whether this depends on lifetime oestrogen exposure, which can have lasting effects on the brain and body even after menopause. Using the UK Biobank sample, we investigated associations between brain characteristics and visceral adipose tissue (VAT) and abdominal subcutaneous adipose tissue (ASAT) in 10,251 post-menopausal females, and assessed whether the relationships varied depending on length of reproductive span (age at menarche to age at menopause). To parse the effects of common genetic variation, we computed polygenic scores for reproductive span. The results showed that higher VAT and ASAT were both associated with higher grey and white matter brain age, and greater white matter hyperintensity load. The associations varied positively with reproductive span, indicating more prominent associations between adipose tissue and brain measures in females with a longer reproductive span. The effects were in general small, but could not be fully explained by genetic variation or relevant confounders. Our findings indicate that associations between abdominal adipose tissue and brain health post-menopause may partly depend on individual differences in cumulative oestrogen exposure during reproductive years, emphasising the complexity of neural and endocrine ageing processes in females.

## Highlights

• Menopause involves decreased oestrogen and higher risk of obesity, which can impact brain health.
• Body composition, oestrogen exposure, and brain health post-menopause is largely unexplored.
• Higher adipose tissue post-menopause is linked to older brain age and WM hyperintensities.
• Associations were stronger in females with longer reproductive spans.
• Body-brain associations post-menopause may partly depend on lifetime oestrogen exposure.

## 1 Introduction

The menopause transition is characterised by decreased circulating oestradiol levels and the cessation of menstrual cycles, marking the end of the reproductive phase (Hall, 2015, Jett et al., 2022, Marlatt et al., 2022). Although many individuals transition through menopause without long-term health issues, this life phase involves higher risk of obesity (Khoudary et al., 2015, Leeners et al., 2017, Lovejoy et al., 2008, Lizcano and Guzmán, 2014) and cardiometabolic diseases (Carr, 2003, Janssen et al., 2008, Pu et al., 2017), which may contribute to the observed post-menopausal risk for neurodegeneration and dementia (Brinton et al., 2015, Jett et al., 2022, Rahman et al., 2019).

The relationships between oestrogen exposure, body composition, and brain health in females are complex and largely unexplored. The menopause transition is linked to an accelerated increase of central fat accumulation (Lizcano and Guzmán, 2014), and abdominal adipose tissue has been associated with higher grey matter (GM) and white matter (WM) brain age (Beck et al., 2022, Beck et al., 2022, Subramaniapillai et al., 2022), WM hyperintensities (WMH) (Arnoldussen et al., 2019, Han et al., 2021, Lampe et al., 2019, Pasha et al., 2017, Park et al., 2018, Vuorinen et al., 2014), and dementia risk (Kiliaan et al., 2014, Tang et al., 2021, Razay et al., 2006, Whitmer et al., 2008). However, in females, adipose tissue also serves as the primary biosynthesis site of oestrogens post-menopause (Steiner and Berry, 2022, Bhardwaj et al., 2019, Kershaw and Flier, 2004, Siiteri, 1987, Simpson, 2003). Since oestradiol is consistently found to exert neuroprotective effects on the pre-menopausal female brain across preclinical and clinical studies (Azcoitia et al., 2019, Barth et al., 2016, Galea et al., 2017, Jacobs and Goldstein, 2018, Merlo et al., 2017, Scott et al., 2012, Zárate et al., 2017), changes in adipose tissue distribution could also involve mechanisms that foster a protective source of oestrogens after menopause (Klosinski et al., 2015, Subramaniapillai et al., 2022). Although the oestrogen levels produced via adipose tissue do not fully compensate for the loss of ovarian oestrogen production (Steiner and Berry, 2022), it is possible that different types of adipose tissue may play diverging roles in female brain health post-menopause.

Studies utilising magnetic resonance imaging (MRI) of the body, which allows for more precise measures of fat distribution than conventional anthropomorphic methods (Borga et al., 2018), demonstrate that visceral adipose tissue (VAT; the fat surrounding the abdominal organs) increases more following menopause than abdominal subcutaneous adipose tissue (ASAT; the fat below the skin) (Lee et al., 2009, Leeners et al., 2017, Lovejoy et al., 2008, Samargandy et al., 2021). Consistent evidence shows that higher midlife VAT in both males and females is associated with lower cortical and total brain volume (Debette et al., 2010, Isaac et al., 2011, Veit et al., 2014), higher WMH load (Anand et al., 2022, Kim et al., 2017, Pasha et al., 2017), and accelerated brain ageing (Zsido et al., 2019), while some studies indicate that ASAT may be significantly less detrimental or even protective for brain volume (Debette et al., 2010, Isaac et al., 2011, Widya et al., 2015, Qi et al., 2021) and WMH load (Kim et al., 2017, Yamashiro et al., 2014), especially in females (Nam et al., 2019). Although midlife adipose tissue levels relate to brain health in both males and females, the known menopause-related changes in body composition (Lizcano and Guzmán, 2014) highlight the need for targeted research into VAT, ASAT, and brain characteristics in post-menopausal females, which have not been examined previously.

In females, levels of oestrogen exposure pre-menopause may influence both brain health and body composition post-menopause, emphasising the complex interactions between neuroendocrine and metabolic processes across the female lifespan. For example, levels of cumulative oestrogen exposure, often assessed by reproductive span (age at menarche to age at menopause; (Fu et al., 2022, Gilsanz et al., 2019, Jett et al., 2022), have been linked to larger GM volumes (Schelbaum et al., 2021), lower WM brain age (Subramaniapillai et al., 2022), and lower dementia risk in older-age samples (Fox et al., 2013, Gilsanz et al., 2019, Gong et al., 2022), although contrasting results have linked a longer reproductive span to increased risk of Alzheimer’s disease (Najar et al., 2020, Geerlings et al., 2001). Age at menarche and menopause are also known to have genetic components (Fernández-Rhodes et al., 2018, Wang et al., 2019, Ruth et al., 2021), but it is unclear how the genetics underlying reproductive span relate to body composition and brain structure (Roa-Díaz et al., 2021). A later age at natural menopause has also been associated with lower risk for post-menopausal abdominal obesity (Zsakai et al., 2015), smaller post-menopausal increase of BMI (Montazeri et al., 2019), and decreased risk for cardiometabolic diseases (Muka et al., 2016, Roa-Díaz et al., 2021, Yang et al., 2017). However, the relationship between these are likely to be bidirectional, as pre-menopausal body composition can influence the timing of natural menopause (Dorjgochoo et al., 2008, Roa-Díaz et al., 2021, Tao et al., 2015, Zhu et al., 2019). Although increasing evidence points to greater lifetime exposure to oestrogens as beneficial for neural and cardiometabolic health, the mechanisms of these long-lasting actions of oestrogens are poorly understood. It is also unclear how cumulative oestrogen exposure during reproductive years interacts with adipose tissue and its post-menopausal oestrogen production to influence brain health at later life stages.

In this study, we investigated associations between different types of abdominal adipose tissue and brain characteristics in 10,251 post-menopausal females, and assessed whether the relationships varied depending on length of reproductive span (age at menarche to age at menopause). Measures of VAT and ASAT were extracted based on body MRI (Linge et al., 2018), and GM- and WM-specific brain age estimates were generated using T1- and diffusion-weighted MRI (dMRI) data, respectively (Voldsbekk et al., 2021). Brain age prediction has emerged as a useful tool for combining a rich variety of brain characteristics into single estimates per individual, providing a reliable proxy of brain integrity and health (Franke et al., 2010, Cole et al., 2019, Beck et al., 2021). Based on recent studies suggesting that tissue-specific age prediction can provide further detail (Beck et al., 2022, de Lange et al., 2020a, Eavani et al., 2018, Voldsbekk et al., 2021), we estimated GM and WM brain age separately. WMH volume derived from T2 fluid-attenuated inversion recovery (FLAIR) images was examined as an additional measure, as a number of studies indicate higher WMH prevalence in females compared to males (Alqarni et al., 2021, Jorgensen et al., 2018, Lohner et al., 2022, Sachdev et al., 2009, Than et al., 2021, Van Den Heuvel et al., 2004) and recent evidence points to sex-specific associations between cardiometabolic risk factors and WMH pathology (Alqarni et al., 2021). Sex differences in WMH prevalence have been observed to primarily emerge after the age of 50 (Wen et al., 2009), which is close to the average age of menopause (51 years, (InterLACE, 2019)), or specifically after the menopause (Jorgensen et al., 2018, Lohner et al., 2022, Than et al., 2021), indicating a link between WMHs and female endocrine ageing processes (Thurston et al., 2016). We used Bayesian linear models to assess relationships between the brain measures and VAT and ASAT, and included interaction terms to test if associations varied depending on reproductive span. To parse the effects of common genetic variation, we also tested for associations between the brain measures and polygenic scores (PGS) for the phenotype reproductive span.

We hypothesised that i) greater levels of abdominal adipose tissue, particularly VAT, would be associated with higher brain age and WMH load, ii) a shorter reproductive span would be associated with higher brain age and WMH load, and iii) the associations between abdominal adipose tissue and brain measures would vary depending on reproductive span, possibly reflecting a protective effect of adipose tissue in females with a shorter reproductive span.

## 2 Methods and materials

### 2.1 Sample characteristics

The sample was drawn from the UK Biobank cohort (www.ukbiobank.ac.uk), and included 20,540 female participants with both T1- and diffusion-weighted MRI data. To ensure a neurologically healthy sample, 1,759 participants with disorders known to affect the brain, including stroke, dementia, and neurodegenerative and psychiatric disorders, were excluded based on ICD10 diagnoses in line with earlier work (Voldsbekk et al., 2021, de Lange et al., 2020) (details are provided in the UK Biobank online resources: http://biobank.ndph.ox.ac.uk/showcase/field.cgi?id=41270). In addition, 160 participants were excluded based on poor-quality MRI data likely due to motion (see Section 2.2), yielding a total of 18,621 participants with T1- and diffusion-weighted MRI data. Out of these, 16,542 participants had data entries across demographic factors, WMH volume, ASAT, VAT, age at menopause, age at menarche, hysterectomy, and bilateral oophorectomy. After removing missing values (NaN, ‘*prefer not to answer*’, ‘*do not know*’), 11,381 were included in the subsequent analyses (missing data = 271 for demographic factors, 629 for WMH volume, 4,329 for age at menopause/menarche, and 1,806 for hysterectomy/oophorectomy, with some participants having missing values across several variables). Participants who had undergone a hysterectomy and/or oophorectomy were excluded (N = 1,010) in order to focus on variation in natural menopause. To avoid outlier-driven results, participants with age at menarche <9 and >17 and age at menopause <39 and >63 were excluded (N = 120, see Section 2.5), yielding a final sample of 10,251. As a cross-check, we also conducted the analyses including all ages at menarche/menopause as well as participants with hysterectomy and/or oophorectomy. Sample demographics are provided in Table 1.

**Table 1:** Sample demographics. Percentage in each group for ethnic background, education, and assessment location. Mean ± standard deviation (SD) and ranges for age, visceral adipose tissue (VAT), abdominal subcutaneous adipose tissue (ASAT), reproductive span, age at menarche, and age at menopause. GCSE = General Certificate of Secondary Education, NVQ = National Vocational Qualification.

**Sample N**		10,251

**Age**	Mean ± SD	63.99 ± 6.63
	Range [years]	48.09–81.49

**Ethnic background**	% White	97.55
	% Black	0.52
	% Mixed	0.42
	% Asian	0.65
	% Chinese	0.34
	% Other	0.52

**Education**	% University/college degree	47.25
	% A levels or equivalent	14.52
	% O levels/GCSE or equivalent	20.26
	% NVQ or equivalent	6.96
	% Professional qualification	5.62
	% None of the above	5.39

**Assessment location**	% Newcastle	27.25
	% Cheadle	58.94
	% Reading	13.81

**VAT**	Mean ± SD	0.95 ± 0.54
	Range	0.04–4.11

**ASAT**	Mean ± SD	2.90 ± 1.24
	Range	0.19–9.60

**Reproductive span [years]**	Mean ± SD	37.95 ± 4.28
	Range	23–51

**Age at menarche [years]**	Mean ± SD	12.99 ± 1.50
	Range	9–17

**Age at menopause [years]**	Mean ± SD	50.94 ± 4.02
	Range	39–63

### 2.2 MRI data acquisition and processing

Information about the UK Biobank data acquisition protocols is available in (Alfaro-Almagro et al., 2018, Miller et al., 2016). Raw T1-weighted MRI data were processed using a harmonised analysis pipeline, including the FreeSurfer (version 5.3) automated surface-based morphometry and subcortical segmentation (Fischl et al., 2002). In line with recent brain age studies (Kaufmann et al., 2019, de Lange et al., 2019, Voldsbekk et al., 2021), we used the standard set of subcortical and cortical summary statistics from FreeSurfer (Fischl et al., 2002), as well as a fine-grained cortical parcellation scheme (Glasser et al., 2016), to extract cortical thickness, area, and volume for 180 regions of interest per hemisphere. This yielded a total set of 1,118 structural brain imaging features (360/360/360 for cortical thickness/area/volume respectively, and 38 for cerebellar/subcortical and cortical summary statistics). All 1,118 features were used as input features in the GM-specific age prediction model (Section 2.3). The brain morphometric data obtained from Freesurfer were residualised with respect to scanning site and intracranial volume using linear models. To remove poor-quality MRI data likely due to motion, participants with Euler numbers (Rosen et al., 2018) ± 4 standard deviations from the mean were excluded (N = 160).

The dMRI data were processed using an optimised diffusion pipeline as described in detail in (Maximov et al., 2019). Metrics derived from diffusion tensor imaging (DTI) (Basser et al., 1994), diffusion kurtosis imaging (DKI) (Jensen et al., 2005), WM tract integrity (WMTI) (Fieremans et al., 2011), and spherical mean technique (SMT) (Kaden et al., 2016, Kaden et al., 2016) were used as input features in the WM-specific age prediction model (Section 2.3), as described in Voldsbekk et al., 2021. The metrics for each diffusion model are listed in Supplementary Information (SI) Section 1. For each metric, WM features were extracted based on John Hopkins University (JHU) atlases for white matter tracts and labels (with 0 thresholding) (Mori et al., 2005), including global mean values and regional measures (Voldsbekk et al., 2021, Beck et al., 2021). The dMRI data were residualised with respect to scanning site using linear models, and passed tract-based spatial statistics (TBSS) post-processing quality control using the YTTRIUM algorithm (Maximov et al., 2021).

Total volume of WMH was derived for each participant based on T2 FLAIR images in combination with T1-weighted data (https://biobank.ndph.ox.ac.uk/showcase/field.cgi?id=25781) using the Brain Intensity Abnormality Classification Algorithm (BIANCA) (Griffanti et al., 2016), which is part of the FMRIB Software Library FSL (Jenkinson et al., 2012). BIANCA is a fully automated tool for segmentation of WMH based on the k-nearest neighbour algorithm, and is documented as a reliable method for WMH segmentation in large cross-sectional cohort studies (Griffanti et al., 2016). The WMH volume measures were log transformed to normalise and stabilise the variance (Veldsman et al., 2020, Wartolowska and Webb, 2021). WMH volume was examined separately, as we were specifically interested in this measure due to the known female prevalence and links to oestradiol levels and adipose tissue. Hence, this feature was not included in the WM brain age estimate.

### 2.3 Brain age prediction

GM- and WM-specific age prediction models were run using the XGBoost regression algorithm (*eXtreme Gradient Boosting*; https://github.com/dmlc/xgboost). XGBoost includes advanced regularisation to reduce overfitting, and has shown superior performance in machine learning competitions (Chen and Guestrin, 2016). Parameters were tuned in a nested cross-validation using 5 inner folds for randomised search, and 10 outer folds for model validation (see https://github.com/amdelange/brainage_women/blob/main/python/run_prediction_model.py for general model setup). Brain age gap (BAG) values were calculated by subtracting chronological age from predicted brain age, providing an estimate of each participant’s brain age relative to their chronological age (Cole and Franke, 2017). To ensure that any associations with the variables of interest were not driven by age-dependence in the predictions (Liang et al., 2019, Smith et al., 2019), chronological age was included as a covariate in all subsequent analyses (de and Cole, 2020, Le et al., 2018).

### 2.4 Abdominal adipose tissue measures

Abdominal adipose tissue measures derived from body MRI were processed by AMRA medical, and accessed via UK Biobank Returned Datasets (Return ID 3666; https://biobank.ndph.ox.ac.uk/ukb/app.cgi?id=6569). The extracted measures included VAT volume, measured within the abdominal cavity, and ASAT volume, measured from the top of the femoral head to the top of the thoracic vertebrae T9, both measured in litres and divided by height squared.

### 2.5 Reproductive span

To calculate reproductive span (age at menopause – age at menarche), we first removed extreme outliers for age at menarche and age at menopause in the sample used for the PGS (see Section 2.6), using median absolute deviation (Leys et al., 2013) with a threshold of 4. The same cut-offs were used in the MRI sample, resulting in a mean reproductive span of 37.95 years ± 4.28 (SD) in the final sample (see Section 2.1 for ages of menarche/menopause removed, and Section 2.8 for cross-checks including all ages at menarche/menopause).

### 2.6 PGS calculations

A genome-wide association study (GWAS) was run on the UK Biobank female cohort (N = 121,620, excluding the MRI sample), using PLINK 2.0 (Chang et al., 2015) with the default additive model, making use of the UK Biobank v3 imputed genetic data, filtering out single nucleotide polymorphisms (SNPs) with a minor allele frequency below 0.001 or failing the Hardy–Weinberg equilibrium test at $p<1.00\times 10^{-9}$. Individuals with known brain disorders as indicated by ICD10 diagnoses (see Section 2.1), previous hysterectomy, and/or oophorectomy, and non-white Europeans were excluded. Linear regressions were run on the variable reproductive span, covarying for age and the first 20 genetic principal components (https://biobank.ndph.ox.ac.uk/showcase/field.cgi?id=22009). PRSice v2 (Choi and O’Reilly, 2019, Euesden et al., 2015) was used to calculate PGS of reproductive span at a p-value threshold of 0.05 for each European individual in the MRI subsample, using PRSice default settings. This includes the removal of the major histocompatibility complex (MHC) (chromosome 6, 26 to 33 Mb) and thinning of SNPs based on linkage disequilibrium and p-value.

### 2.7 Statistical analyses

In line with guidelines for reporting Bayesian analyses (Sung et al., 2005, Kruschke, 2021), we list the statistical software used, define our priors, describe the statistical models used, and quote the results using central tendencies (mean, median, mode) and credible intervals. To test for associations between the brain measures and adipose tissue (VAT and ASAT) and their interaction with reproductive span, we ran Bayesian multiple linear models using the Bayesian Model-Building Interface (Bambi) package (Capretto et al., 2022) in Python 3.7.6 (https://pypi.org/project/bambi). All variables were standardised (subtracting the mean and dividing by the standard deviation) prior to analyses. Four chains, with 2000 samples each, were estimated. In the sampling process, the first 1000 samples served as burn-ins to identify the region of best-fitting values in the parameter space. Weakly informative normal priors with μ=0 and σ=2.5 were generated for all model terms by loosely scaling them to the standardised data (Bambi default) (Capretto et al., 2022). The model results for each association are described by the mean and the 95% highest density interval (HDI) of its posterior distributions. The mean represents the central tendency of the association, while there is a 95% probability that the true value lies within the HDI (see e.g. Hespanhol et al., 2019). Median and mode central tendencies are also provided.

Models were run with VAT and ASAT separately due to high correlation between the variables (r = 0.77). The models included brain measure (GM BAG, WM BAG, or WMH vol) as the dependent variable, adipose tissue volume (VAT or ASAT) × reproductive span as independent variables, and age as a covariate:

(1) Brainmeasure∼adiposetissuevolume×reproductivespan+age.

To test for effects of common genetic variation, we calculated the phenotypic variance explained by the reproductive span PGS, tested for main effects of PGS on the brain measures, and re-ran the main analyses including the PGS as a covariate.

### 2.8 Sensitivity analyses

To account for potential confounding factors that could influence brain structure, adipose tissue levels, and/or reproductive span, the models were rerun with the following covariates (in addition to age): health factors including diabetes status (Cole, 2020, Peters et al., 2014), hypertension (Fuchs and Whelton, 2020, Newby et al., 2022) and a lifestyle score which was computed by adding one point per unhealthy lifestyle factor (physical activity level, intake of fruits, vegetables, oily fish and red meats, sleep duration, television viewing time, current and past smoking status, and alcohol use) (Foster et al., 2018), female-specific factors including number of previous childbirths (de Lange et al., 2019), hormone replacement therapy use (user versus never user) (Hogervorst et al., 2000, Maki et al., 2011), and oral contraceptive use (user versus never user) (De Bondt et al., 2013), and socioeconomic factors including educational level (Fotenos et al., 2008, Meng and D’arcy, 2012, Walhovd et al., 2022) and ethnic background (Goff, 2019). We first included all covariates in one model, and then in three separate models including i) the health factors, ii) the female-specific factors, and iii) the socioeconomic factors, to test for specific influences of these potential covariates on the results. Furthermore, we repeated the analyses excluding subjects with a BMI >40 (N excluded = 117), as these values may indicate morbid obesity and risk for serious health complications (Jarolimova et al., 2013). To account for potential uncertainties related to self-reporting of age at menarche decades later (Cooper et al., 2006, Must et al., 2002), the models were also conducted using age at menopause instead of reproductive span. Finally, we repeated the analyses without excluding any participants with outlier values for ages at menarche/menopause or hysterectomy and/or oophorectomy.

## 3 Results

### 3.1 Associations with GM/WM BAG and WMH volume

Table 2 shows the age prediction accuracy of the GM- and WM-based models, respectively.

**Table 2:** Age prediction accuracy for the models based on grey matter (GM) and white matter (WM) features, respectively. Model accuracy is measured by R^2^ (proportion of variance in age explained), root mean square error (RMSE), mean absolute error (MAE), and Pearson’s correlations (*r*) between predicted and chronological age. R^2^, RMSE, and MAE are averaged across folds, providing the mean ± standard error of each performance measure. CI = confidence interval.

Model	R^2^	RMSE	MAE	*r* [95% CI]	*p*
GM	0.53±0.015	5.02±0.069	3.99±0.074	0.73[0.72,0.74]	<0.0001
WM	0.60±0.021	4.65±0.089	3.74±0.086	0.77[0.77,0.78]	<0.0001

**Fig. 1:**
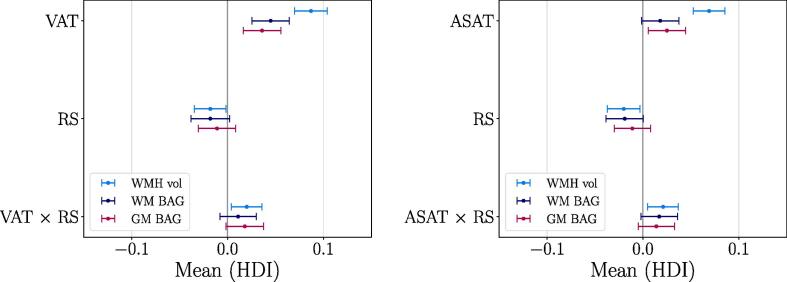
Associations between visceral adipose tissue (VAT), abdominal subcutaneous adipose tissue (ASAT), reproductive span (RS) and brain measures. The points show the means of the posterior distributions for the associations, with error bars indicating the 95% highest density intervals (HDI). Higher VAT and ASAT were associated with higher GM/WM BAG and WMH volume. Shorter RS was associated with higher GM/WM BAG and WMH volume, and the relationships between VAT and ASAT and the brain measures varied positively with RS as indicated by the interaction terms. As the HDIs for the RS associations and interactions approach or overlap with zero, these results are not fully conclusive. GM = grey matter, WM = white matter, BAG = brain age gap, WMH vol = white matter hyperintensity volume.

**Fig. 2:**
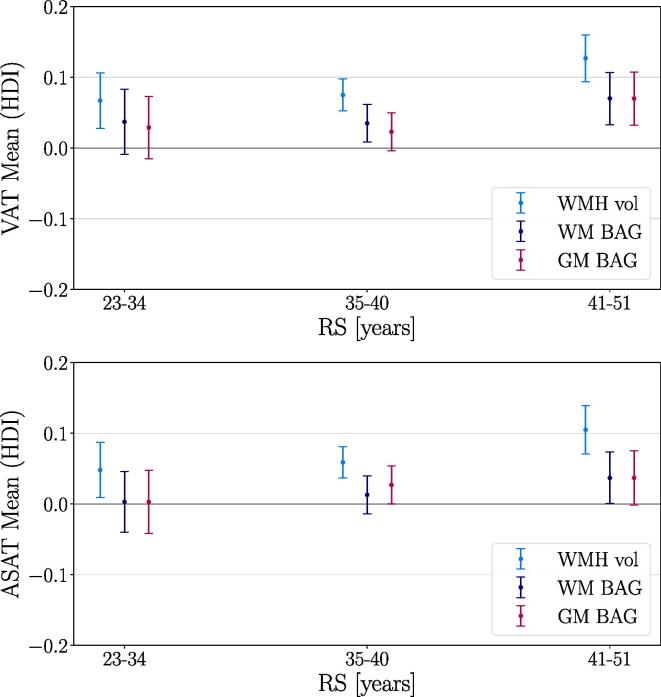
Associations between visceral adipose tissue (VAT) and abdominal subcutaneous adipose tissue (ASAT) and brain measures, estimated in bins of reproductive span (RS) to illustrate the interaction effects observed in Fig. 1. In females with a longer RS, higher VAT and ASAT was slightly more positively associated with GM/WM BAG and WMH vol than in subjects with a shorter RS. Note that the continuous RS variable was used in the analyses (Eq. 1), and the bins are created only to visualise the direction of the interaction. The points show the means of the posterior distributions for the associations, with error bars indicating the 95% highest density intervals (HDI). The mean ± SD for RS was 37.95 ± 4.28 years, with bins including 1,914, 5,564, and 2,772 participants with a RS between 23–34, 35–40, and 41–51 years, respectively. VAT = visceral adipose tissue, ASAT = abdominal subcutaneous adipose tissue, GM = grey matter, WM = white matter, BAG = brain age gap, WMH vol = white matter hyperintensity volume.

**Fig. 3:**
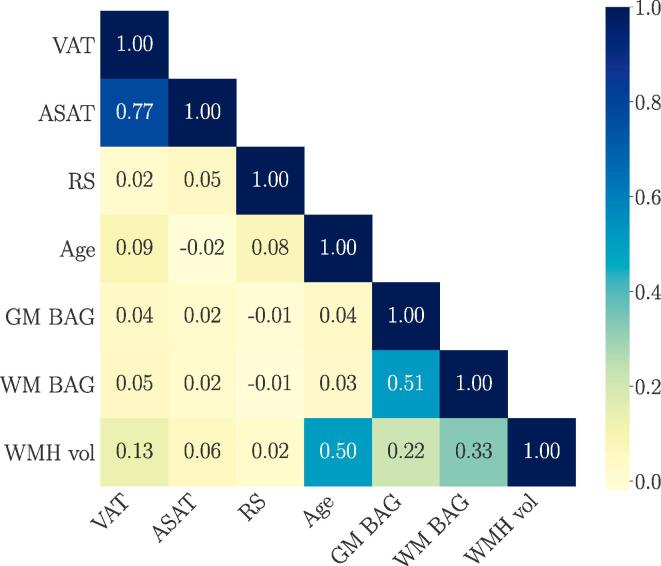
Correlations between visceral adipose tissue (VAT), abdominal subcutaneous adipose tissue (ASAT), reproductive span (RS), age, grey matter (GM) and white matter (WM) brain age gap (BAG), and white matter hyperintensity volume (WMH vol).

Fig. 1 and Table 3 show the associations of the brain measures with VAT, ASAT, reproductive span, and the interaction terms (see Eq. 1), as described by the mean and the 95% HDI of their posterior distributions. Table 3 also includes mode and median values, which were highly similar to the means across associations. SI Fig. 1 shows the posterior distributions from the model including VAT, RS, and GM BAG for illustration. Higher VAT and ASAT were associated with higher BAG (i.e., older brain age relative to chronological age) and higher WMH volume. A shorter reproductive span was related to higher GM/WM BAG and WMH volume. This is indicative of a negative relationship between reproductive span and the brain measures, but the results are not fully conclusive as the upper HDIs approach or overlap with zero (see Table 3). As a cross-check, we measured the main effects of ASAT, VAT, and reproductive span on the brain measures in separate models that did not include the interaction term. The associations were consistent, as shown in SI Fig. 2. The relationships between VAT and ASAT and the brain measures varied positively with reproductive span based on the HDI mean values, such that longer reproductive spans and higher VAT and ASAT were associated with higher BAG and WMH load. However, these interactions are not fully conclusive as the lower HDIs approach or overlap with zero (see Table 3). To illustrate the interaction effects, Fig. 2 shows the VAT and ASAT associations with the brain measures in bins of reproductive span length. Fig. 3 shows the correlations between VAT and ASAT, reproductive span, and the brain measures.

**Table 3:** Means and highest density intervals (HDIs) of the posterior distributions for each Bayesian regression model, in addition to mode and median values for each association. VAT = visceral adipose tissue, ASAT = abdominal subcutaneous adipose tissue, RS = reproductive span, GM = grey matter, WM = white matter, BAG = brain age gap, WMH vol = white matter hyperintensity volume.

Brain measures	Term	Mean	Mode	Median	HDI 2.5%	HDI 97.5%
GM BAG	VAT	0.036	0.036	0.036	0.017	0.056
	RS	−0.011	−0.013	−0.011	−0.032	0.007
	VAT × RS	0.018	0.018	0.018	−0.002	0.037

WM BAG	VAT	0.045	0.044	0.045	0.025	0.064
	RS	−0.018	−0.017	−0.018	−0.038	0.002
	VAT × RS	0.011	0.012	0.012	−0.008	0.030

WMH vol	VAT	0.087	0.086	0.087	0.071	0.105
	RS	−0.018	−0.017	−0.018	−0.033	0.000
	VAT × RS	0.020	0.020	0.020	0.004	0.036

GM BAG	ASAT	0.025	0.022	0.025	0.005	0.044
	RS	−0.011	−0.012	−0.011	−0.030	0.008
	ASAT × RS	0.014	0.015	0.014	−0.005	0.033

WM BAG	ASAT	0.018	0.019	0.018	−0.003	0.036
	RS	−0.019	−0.018	−0.018	−0.038	0.001
	ASAT × RS	0.017	0.016	0.017	−0.002	0.036

WMH vol	ASAT	0.069	0.069	0.069	0.053	0.086
	RS	−0.020	−0.018	−0.019	−0.036	−0.002
	ASAT × RS	0.021	0.021	0.021	0.005	0.037

### 3.2 Reproductive span PGS

**Fig. 4:**
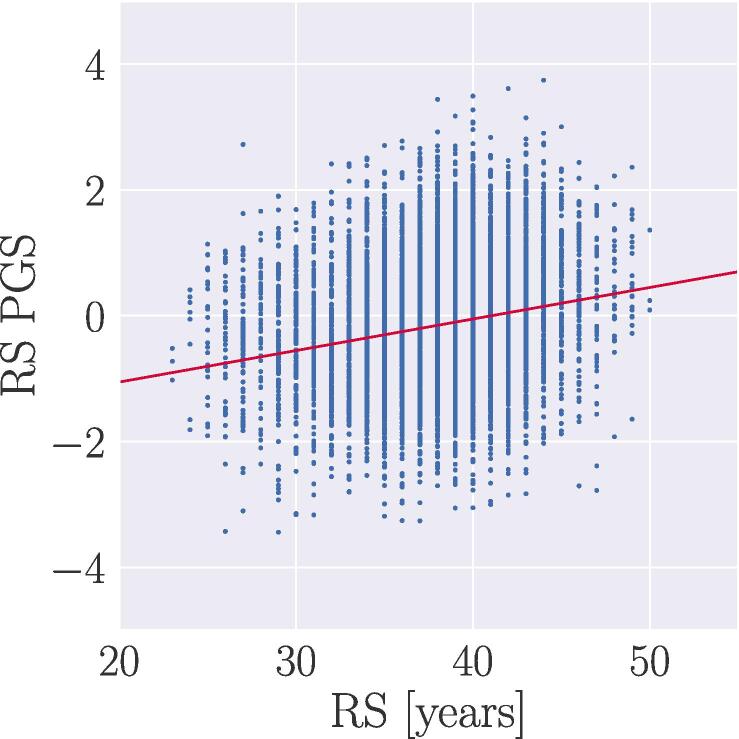
Reproductive span in years (x-axis) versus polygenic score (PGS) for reproductive span (y-axis), based on a linear regression adjusting for age. The adjusted $R^{2}$ value was 0.045, with a β (slope) value of 0.05±0.003 (standard error).

To measure the phenotypic variance explained by the reproductive span PGS, we ran a linear regression for PGS and reproductive span in years to calculate $R^{2}$, adjusting for age (Choi et al., 2020). The adjusted $R^{2}$ value was 0.045, with a β value of 0.05±0.003 (standard error) for the PGS, as shown in Fig. 4. The PGS scores showed no associations with the brain measures (SI Fig. 3), and the correlations of RS PGS with VAT and ASAT were r = −0.02 and −0.01, respectively (see SI Fig. 4 for correlation matrix). The associations between VAT and ASAT, reproductive span, and the brain measures persisted when partialling out polygenic scores, as shown in SI Fig. 5 and SI Table 1.

### 3.3 Sensitivity analyses

The sensitivity analyses showed that when including the additional covariates specified in Section 2.8 in a single model, the associations showed a pattern consistent with the main results, but with minor shifts towards zero for the main effects of both adipose tissue types and reproductive span on brain measures. The three separate covariate models (SI Fig. 7) showed that the positive associations between adipose tissue and the brain measures were slightly weaker when including the health factors (diabetes, hypertension, and the lifestyle score), and the negative associations between reproductive span and the brain measures were slightly weaker when including the female-specific factors (number of previous childbirths, hormone replacement therapy use and oral contraceptive use). When including only the socioeconomic factors (ethnic background and educational level), the associations were highly consistent with the main results. The results remained consistent with the main results when excluding subjects with a BMI above 40 (SI Fig. 8), when repeating the models using age at menopause instead of reproductive span (SI Fig. 9), and when including all ages at menarche/menopause as well as participants with hysterectomy and/or oophorectomy (SI Fig. 10).

## 4 Discussion

This study examined the associations between different types of abdominal adipose tissue (VAT and ASAT), reproductive span, and brain characteristics (GM/WM BAG and WMH volume) in a large sample of post-menopausal females. In summary, greater VAT and ASAT were both associated with higher GM/WM BAG (older brain age relative to chronological age) and higher WMH volume. Based on the HDI mean values, a shorter reproductive span was related to higher GM/WM BAG and WMH volume, and the associations between abdominal adipose tissue and brain measures varied positively with reproductive span, potentially indicating more prominent associations in females with greater levels of lifetime oestrogen exposure. The effects were in general small, but could not be fully explained by genetic variation or relevant confounders, and further studies are needed to draw conclusions.

The associations between abdominal adipose tissue and brain characteristics are consistent with previous studies linking elevated adipose tissue to older brain age (Beck et al., 2022, Subramaniapillai et al., 2022), lower brain volume (Cho et al., 2021, Debette and Markus, 2010, Gurholt et al., 2021, Isaac et al., 2011, Veit et al., 2014), and higher WMH load (Arnoldussen et al., 2019, Lampe et al., 2019, Park et al., 2018, Vuorinen et al., 2014). The relationships were more prominent for WMH volume compared to GM and WM BAG (about 3 standard deviations higher, see Fig. 1), indicating that the WMH volume measures from FLAIR images may represent a particularly sensitive measure. Although WMH are likely to also influence WM diffusion measures (Raghavan et al., 2021), this finding may suggest an increased risk for white matter lesions in post-menopausal females with elevated abdominal adipose tissue. Inflammation has been proposed as a key factor linking central adiposity and WMH load (Lampe et al., 2019), and weight gain during the menopause transition involves a heightened inflammatory state (Lee et al., 2009, McCarthy and Raval, 2020). Inflammation linked to changes in hormone levels and body composition may thus be a mechanistic explanation for the higher risk of WMH in post-menopausal females (Fatemi et al., 2018, Sachdev et al., 2009, Than et al., 2022, Wen et al., 2009). However, the relationship between changes in adipose tissue and WM lesions within the shorter perimenopausal time window remains to be investigated.

The associations with brain characteristics showed similar patterns for the two abdominal adipose tissue types (see Fig. 1). Although VAT and ASAT may have distinct anatomical, cellular, molecular, physiological, clinical, and prognostic correlates (Ibrahim, 2010, Kwok et al., 2016), the high correlation between them (>0.7) in the current study indicates that these measures shared a relatively large degree of overlapping information. However, other studies have specifically linked midlife VAT to more adverse effects on brain structure compared to ASAT (Debette et al., 2010, Isaac et al., 2011, Widya et al., 2015, Qi et al., 2021). One possible explanation for this arises from a biopsy study, which showed changes in adipose tissue phenotypes across the menopause transition (Abildgaard et al., 2021). Specifically, these changes were linked to metabolic dysfunction in both VAT and ASAT post-menopause (Abildgaard et al., 2021), which could potentially contribute to detrimental effects of both tissue types on brain structure. However, longitudinal studies assessing changes in adipose tissue phenotype and brain health across the menopause transition and beyond are needed to draw causal conclusions.

Although adipose tissue is the primary biosynthesis site of oestrogens post-menopause (Bhardwaj et al., 2019), we found no direct evidence towards neuroprotective effects of certain adipose tissue types. We did however observe that the associations of VAT and ASAT with the brain measures were more prominent in females with a longer reproductive span. Although these results were not fully conclusive (see Section 3.1), this finding could indicate that a combination of higher levels of adipose tissue and greater exposure to oestrogens may constitute a risk of adverse brain health (see e.g. Brinton, 2005, Brinton, 2008). Alternatively, these findings could indicate that with earlier decline of ovarian hormone production, higher levels of adipose tissue may be less detrimental due to beneficial oestrogen production via adipose tissue. However, it is unclear how individual variation in oestrogen exposure pre-menopause may influence associations between adipose tissue and brain health post-menopause, and how this may relate to circulating oestrogen levels, which were not available at the time of the brain and body MRI scans. Although oestradiol measures were available from baseline in a smaller subsample, it can take several years for oestradiol levels to stabilise following menopause (Randolph Jr et al., 2011), and these measurements may be influenced by certain types of hormone replacement therapy (Waaseth et al., 2008) as well as lifestyle factors (Wiggs et al., 2021). Due to the time window between assessments, changes in both oestradiol and adipose tissue levels could occur, limiting any firm conclusions based on these measures. Future studies should target both current and previous oestrogen levels to clarify the links between adipose tissue, oestrogen exposure, and brain characteristics, and ideally measure how changes in hormones and body fat link to brain health across the menopause transition.

Our results further indicated associations between a shorter reproductive span and higher GM/WM BAG and WMH volume independent of abdominal adipose tissue levels. Although these effects were not fully conclusive, the directions of the associations align with previous studies showing beneficial effects of a longer reproductive span on a number of brain health markers (Georgakis et al., 2016, Schelbaum et al., 2021, Subramaniapillai et al., 2022, Zeydan et al., 2019) and dementia risk (Gong et al., 2022). In line with previous studies (Day et al., 2015, Day et al., 2017, Zhao et al., 2021) we found an association between polygenic and phenotypic variance in reproductive span, but we found no notable associations between PGS, adipose tissue measures and brain measures, nor did PGS alter the interactions when included as a covariate. This suggests that the observed associations may be driven by factors such as oestrogen exposure rather than common genetic variation. While it is plausible that longer-term exposure to oestrogens pre-menopause may have lasting effects on brain health beyond the menopause transition (Schelbaum et al., 2021), the observed effects were small, which may explain why other studies have found no association between reproductive span length and brain characteristics (Prince et al., 2022). Proxies of lifetime oestrogen exposure also vary across studies (de Lange et al., 2020, Fox et al., 2013, Zhao et al., 2021), and factors such as duration of hormone replacement/contraceptive use and time spent breastfeeding are likely to influence cumulative oestrogen exposure across the female lifespan (Barth and de Lange, 2020). When including available covariates (hormone replacement therapy and oral contraceptive use, and previous pregnancies), we observed these to slightly moderate the associations between reproductive span and the brain measures, indicating that a range of female-specific factors relate to brain health in line with previous studies (Gong et al., 2022, de Lange et al., 2019).

Whether brain characteristics are influenced by higher abdominal adipose tissue generally or by its typical increase during the menopause transition is unclear. For example, a longitudinal study found that it was the change in BMI over a 20-year period spanning from pre- to post-menopause that predicted GM volume in 48 females (Soreca et al., 2009). Recent studies also point towards a biphasic association between BMI and dementia (Kivimäki et al., 2018, Pedditizi et al., 2016). For example, while midlife obesity predicts risk for dementia (Albanese et al., 2017, Floud et al., 2020, Pedditizi et al., 2016), the prevalence of dementia has been found to be higher in underweight than in normal weight or overweight females (Dong et al., 2022). Prodromal stages of neurodegenerative diseases can involve weight loss as a result of disrupted brain function and dietary changes (Floud et al., 2020, Gu et al., 2014), and longitudinal studies targeting early markers of neurodegeneration may further the understanding of changes in body weight, brain health, and dementia risk in females.

Also yet to be elucidated are the mechanisms underlying the associations between adipose tissue, oestrogen exposure, and brain characteristics, which are likely multifactorial and interactive. Factors such as elevated inflammatory markers have been associated with increased adipose tissue levels (Aguilar-Valles et al., 2015, Miller and Spencer, 2014), even specifically during the menopause transition (Lee et al., 2009), as well as decreasing oestrogens (McCarthy and Raval, 2020), brain atrophy (Luo et al., 2022), and dementia risk (Heneka et al., 2015, Ransohoff, 2016). Biological markers of obesity, such as lipid profile (Anstey et al., 2017, Reitz, 2012), glucose (Crane et al., 2013), HbA1c (Ramirez et al., 2015), leptin (Zeki Al Hazzouri et al., 2013), and Vitamin B12 (Lauer et al., 2022), may also influence associations between adipose tissue and brain health, and contribute to risk of comorbidities such as type II diabetes and hypertension, which are known to impact neural and cardiometabolic health (Cole, 2020, Fuchs and Whelton, 2020, Newby et al., 2022, Peters et al., 2014).

Importantly, sex differences have been observed for the aetiology and progression of cardiometabolic risk factors (Gerdts and Regitz-Zagrosek, 2019, Yoshida et al., 2022) and their relation to the brain (Alqarni et al., 2021, Subramaniapillai et al., 2021, Subramaniapillai et al., 2022, Than et al., 2022), illustrating the critical need for sex-specific studies (Miller and Spencer, 2014, Shansky and Murphy, 2021, Taylor et al., 2019). Further research is necessary to understand the complex interplay of mechanisms that contribute to risk for cardiometabolic and neurodegenerative diseases in post-menopausal females (Christensen and Pike, 2015), and how preventive measures such as physical exercise and hormone replacement therapy can be optimised to moderate risk.

In conclusion, our findings indicate that higher levels of both visceral and abdominal subcutaneous adipose tissue are associated with higher brain age and WMH volume in post-menopausal females. These associations may partly depend on individual differences in cumulative oestrogen exposure, and future studies should aim to disentangle the complex relationships between oestrogen exposure, adipose tissue, and brain health both across the menopause transition and beyond. As the menopause transition involves an accelerated increase of central fat accumulation, further research into mechanisms and risks is pertinent to facilitate preventive interventions that can reduce the risk of adverse brain health post-menopause.

## 5 Data availability statement

The data that support the findings of this study are available through the UK Biobank data access procedures (https://www.ukbiobank.ac.uk/researchers).

## CRediT authorship contribution statement

**Louise S. Schindler:** Conceptualization, Methodology, Formal analysis, Visualization, Writing – original draft. **Sivaniya Subramaniapillai:** Conceptualization, Methodology, Writing – review & editing. **Claudia Barth:** Methodology, Writing – review & editing. **Dennis van der Meer:** Methodology, Formal analysis, Writing – review & editing. **Mads L. Pedersen:** Methodology, Visualization, Writing – review & editing. **Tobias Kaufmann:** Methodology, Writing – review & editing. **Ivan I. Maximov:** Writing – review & editing. **Jennifer Linge:** Methodology, Writing – review & editing. **Olof Dahlqvist Leinhard:** Methodology, Writing – review & editing. **Dani Beck:** Writing – review & editing. **Tiril P. Gurholt:** Writing – review & editing. **Irene Voldsbekk:** Writing – review & editing. **Sana Suri:** Writing – review & editing. **Klaus P. Ebmeier:** Writing – review & editing, Project administration. **Bogdan Draganski:** Writing – review & editing, Project administration. **Ole A. Andreassen:** Writing – review & editing, Project administration. **Lars T. Westlye:** Methodology, Writing – review & editing, Project administration. **Ann-Marie G. de Lange:** Conceptualization, Methodology, Formal analysis, Visualization, Writing – original draft, Project administration.
