# Supplementary material for: Associations between abdominal adipose tissue, reproductive span, and brain characteristics in post-menopausal women
Source: Neuroimage Clin. 2022 Oct 26;36:103239. doi: 10.1016/j.nicl.2022.103239 (PMC9668664; doi:10.1016/j.nicl.2022.103239)
Supplement: Supplementary data [file mmc1.pdf]

Supplementary Information (SI)

1. Features included in the age prediction models

In line with our previous studies (Kaufmann et al., 2019; Voldsbekk et al., 2021), the T1-weighted features included the classic set of subcortical and cortical summary statistics from FreeSurfer (Fischl et al., 2002), and cortical thickness/area/volume extracted based on a fine-grained cortical parcellation atlas described in detail in (Glasser et al., 2016). The diffusion model metrics included mean diffusivity (MD), fractional anisotropy (FA), axial diffusivity (AD), and radial diffusivity (RD) for DTI (Basser et al., 1994), mean kurtosis (MK), axial kurtosis (AK), and radial kurtosis (RK) for the DKI (Jensen et al., 2005), axonal water fraction (AWF), extra-axonal axial diffusivity (axEAD), and extra-axonal radial diffusivity (radEAD) for WMTI (Fieremans et al., 2011), and intra-neurite volume fraction (INVF), extra-neurite mean diffusivity (exMD), and extra-neurite radial diffusivity (exRD) for SMT (Kaden et al., 2016).

2. Posterior distribution plots for the model including VAT, RS and GM BAG

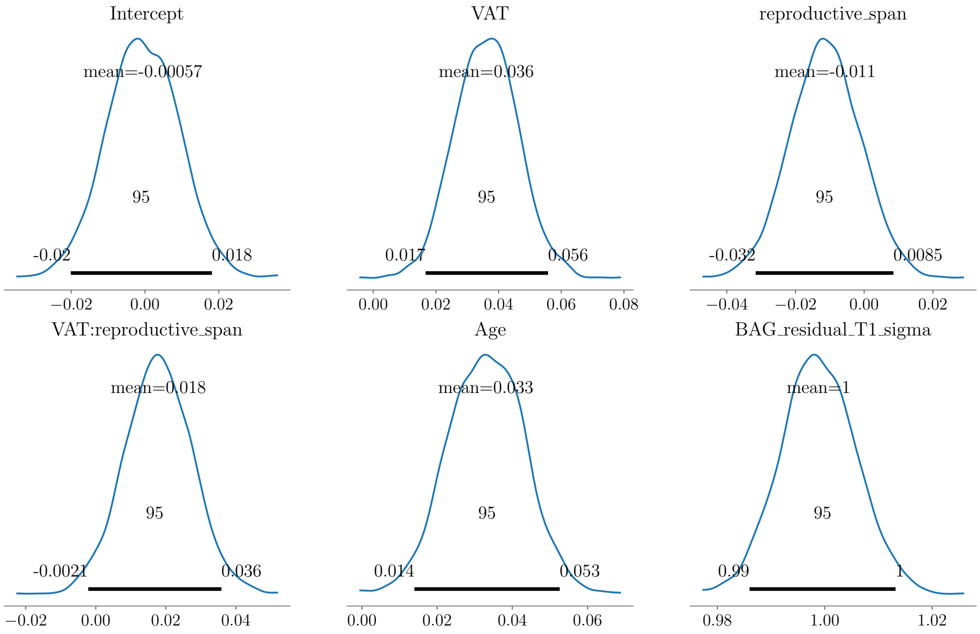

SI Figure 1: Posterior distribution plots of the model including visceral adipose tissue (VAT), reproductive span, and gray matter brain age gap (BAG), with the 95% highest density intervals (HDIs) indicated by the black horizontal bars. The intercept, main effects (VAT, reproductive span, and age as a covariate), interaction (VAT:reproductive span), and error term (BAG_residual_T1_sigma) are shown. The HDI distributions for all other models have the same form, please see Table 3 in the main manuscript for summary statistics.

3. Main effects of VAT, ASAT, and RS from models without interaction terms

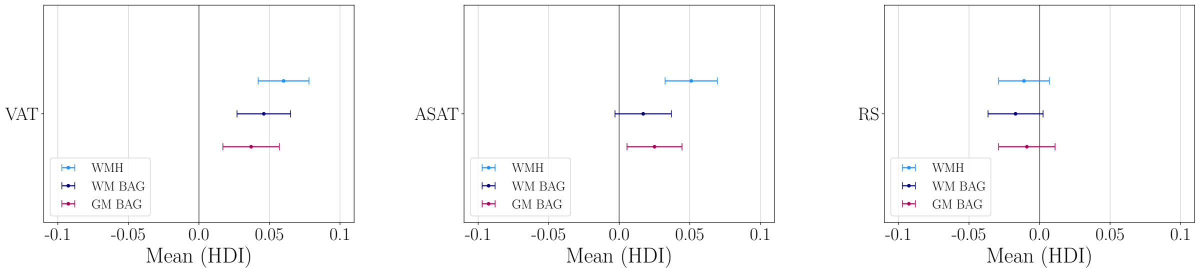

SI Figure 2: Associations between brain measures and visceral adipose tissue (VAT), abdominal subcutaneous adipose tissue (ASAT), and reproductive span (RS) from models without the interaction term: *Brain measure* $\sim$ *VAT/ASAT/RS* + *Age*. The points show the means of the posterior distributions for the associations, with error bars indicating the 95% highest density intervals (HDI). GM = grey matter, WM = white matter, BAG = brain age gap, WMH vol = white matter hyperintensity volume.

4. Reproductive span polygenic scores (PGS)

As shown in SI Figure 3, there were no associations between PGS for reproductive span and the brain measures. As shown in SI Figure 4, the correlations of RS PGS with VAT and ASAT were r = -0.02 and -0.01, respectively, and the associations between VAT and ASAT, reproductive span, and the brain measures persisted when partialling out PGS for reproductive span, as shown in SI Figure 5 and SI Table 1.

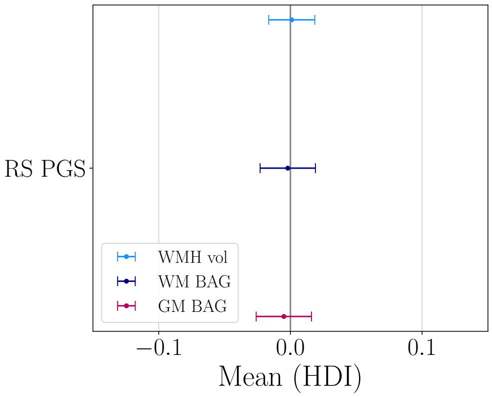

SI Figure 3: Associations between polygenic score for reproductive span (RS PGS) and brain measures, adjusting for age. The points show the means of the posterior distributions for the associations, with error bars indicating the 95% highest density intervals (HDI). N participants with complete data on all variables including PGS = 8,677. GM = grey matter, WM = white matter, BAG = brain age gap, WMH vol = white matter hyperintensity volume.

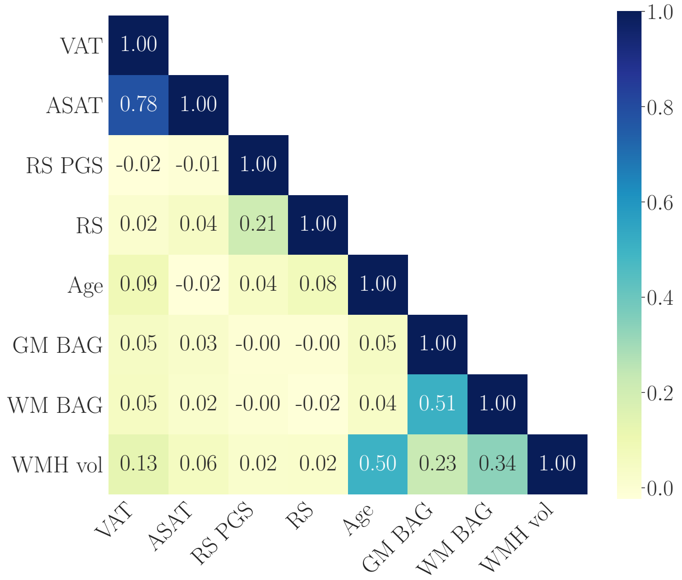

SI Figure 4: Correlations between visceral adipose tissue (VAT), abdominal subcutaneous adipose tissue (ASAT), polygenic score for reproductive span (RS PGS), reproductive span (RS), age, grey matter (GM) and white matter (WM) brain age gap (BAG), and white matter hyperintensity volume (WMH vol). N participants with complete data on all variables = 8,677.

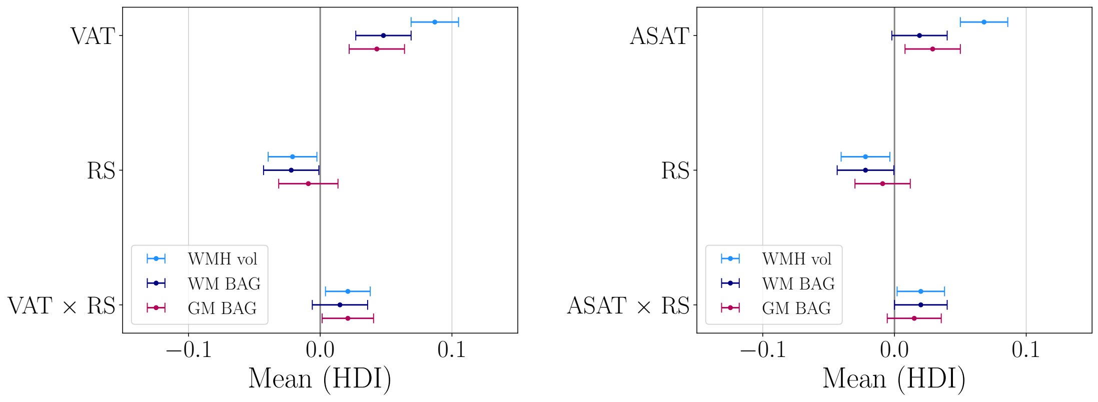

SI Figure 5: Associations between brain measures and visceral adipose tissue (VAT), abdominal subcutaneous adipose tissue (ASAT), reproductive span (RS) and the interaction terms, adjusting for age and polygenic score for reproductive span (RS PGS). The points show the means of the posterior distributions for the associations, with error bars indicating the 95% highest density intervals (HDI). N participants with complete data on all variables including PGS = 8,677. GM = grey matter, WM = white matter, BAG = brain age gap, WMH vol = white matter hyperintensity volume.

SI Table 1: Means and highest density intervals (HDIs) of the posterior distributions for each Bayesian regression model, adjusting for age and polygenic score for reproductive span (RS PGS). N participants with complete data on all variables including PGS = 8,677. VAT = visceral adipose tissue, ASAT = abodminal subcutaneous adipose tissue, RS = reproductive span, GM = grey matter, WM = white matter, BAG = brain age gap, WMH vol = white matter hyperintensity volume.

Brain measures	Term	Mean	HDI 2.5%	HDI 97.5%
GM BAG	VAT	0.043	0.023	0.065
	RS	-0.009	-0.033	0.012
	VAT × RS	0.021	0.003	0.042
WM BAG	VAT	0.048	0.027	0.069
	RS	-0.022	-0.042	-0.000
	VAT × RS	0.015	-0.006	0.036
WMH vol	VAT	0.087	0.068	0.104
	RS	-0.021	-0.041	-0.004
	VAT × RS	0.021	0.005	0.039
GM BAG	ASAT	0.029	0.007	0.049
	RS	-0.009	-0.031	0.011
	ASAT × RS	0.015	-0.005	0.036
WM BAG	ASAT	0.019	-0.001	0.041
	RS	-0.022	-0.043	-0.000
	ASAT × RS	0.020	0.000	0.040
WMH vol	ASAT	0.068	0.049	0.085
	RS	-0.022	-0.041	-0.004
	ASAT × RS	0.020	0.002	0.038

5. Sensitivity analyses

5.1. Additional covariates

Sensitivity analyses included re-running the model with the following covariates: health factors including diabetes status, hypertension, and a lifestyle score consisting of physical activity, smoking, alcohol use, sleep duration, and diet, female-specific factors including number of previous childbirths, hormone replacement therapy use (user versus never user), and oral contraceptive use (user versus never user), and socioeconomic factors including ethnic background and educational level, in addition to age (see manuscript section 2.8) in one model. As shown in SI Figure 6, the associations showed a pattern consistent with the main results, but with minor shifts towards zero for the main effects of both adipose tissue types and reproductive span on brain measures. The three separate covariate models (SI Figure 7) showed that the associations between adipose tissue and the brain characteristics were slightly moderated when including the health factors (diabetes, hypertension, and the lifestyle score), and the associations between reproductive span and brain the brain measures were slightly moderated by including the female-specific factors (number of previous childbirths, hormone replacement therapy use and oral contraceptive use). When including only the socioeconomic factors (ethnic background and educational level), the associations were highly consistent with the main results.

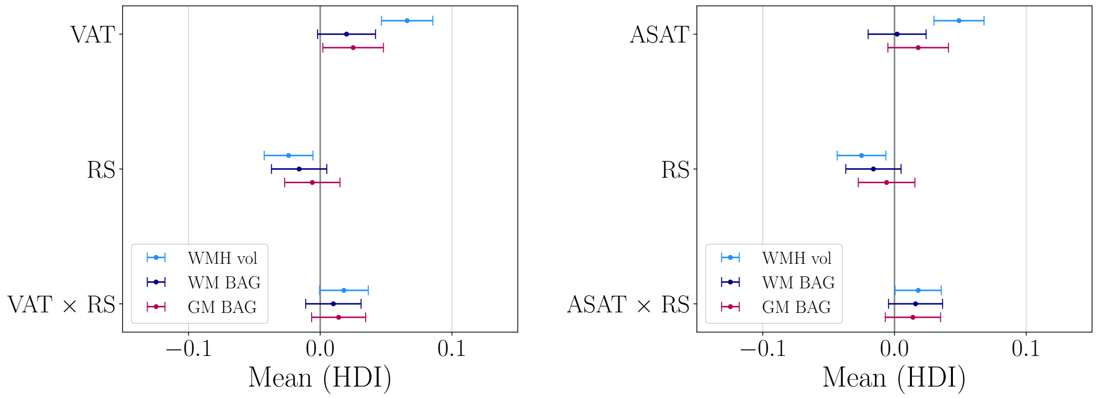

SI Figure 6: Associations between brain measures and visceral adipose tissue (VAT), abdominal subcutaneous adipose tissue (ASAT), and reproductive span (RS) when including all additional covariates as specified above. The points show the means of the posterior distributions for the associations, with error bars indicating the 95% highest density intervals (HDI). N participants with complete data on all variables = 8,272. GM = grey matter, WM = white matter, BAG = brain age gap, WMH vol = white matter hyperintensity volume.

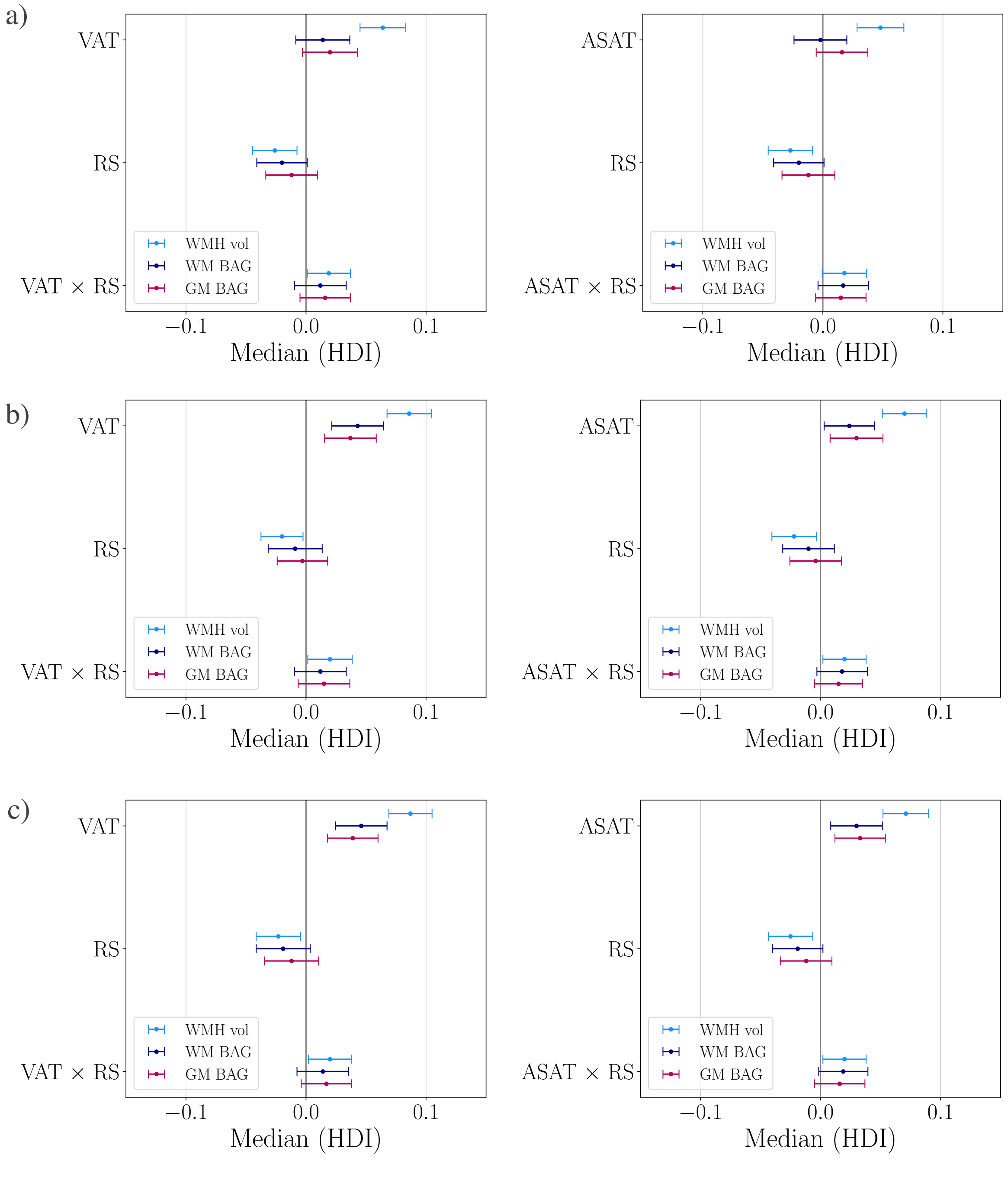

SI Figure 7: Associations between brain measures and visceral adipose tissue (VAT), abdominal subcutaneous adipose tissue (ASAT), and reproductive span (RS) when including different groups of covariates, in addition to age. a) Covariates included hypertension, diabetes status and a lifestyle score (specified in 5.1). b) Covariates included number of previous childbirths, hormone replacement therapy use (user versus never user), and oral contraceptive use (user versus never user). c) Covariates included ethnic background and educational level. The points show the means of the posterior distributions for the associations, with error bars indicating the 95% highest density intervals (HDI). N participants with complete data on all variables = 8,272. GM = grey matter, WM = white matter, BAG = brain age gap, WMH vol = white matter hyperintensity volume.

5.2. *Exclusion of participants with BMI above 40*

Further sensitivity analyses included re-running the models after exclusion of participants with a BMI over 40. As shown in SI Figure 8, the results were relatively consistent with the main analyses.

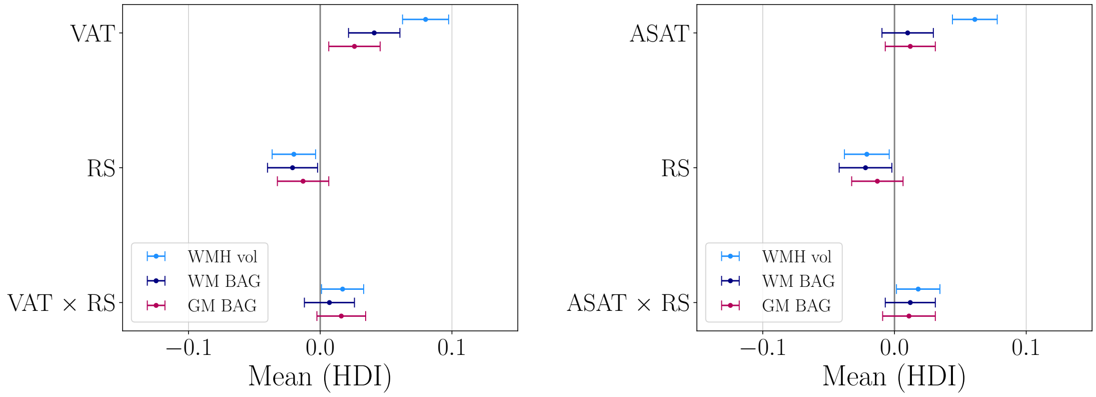

SI Figure 8: Associations between brain measures and visceral adipose tissue (VAT), abdominal subcutaneous adipose tissue (ASAT), and reproductive span (RS) after exclusion of participants with BMI >40. The points show the means of the posterior distributions for the associations, with error bars indicating the 95% highest density intervals (HDI). N participants with complete data on all variables = 10,134. GM = grey matter, WM = white matter, BAG = brain age gap, WMH vol = white matter hyperintensity volume.

5.3. *Age at menopause as independent variable*

When re-running the models with age at natural menopause as an independent variable instead of reproductive span, the associations and interactions were consistent with the main results as shown in SI Figure 9.

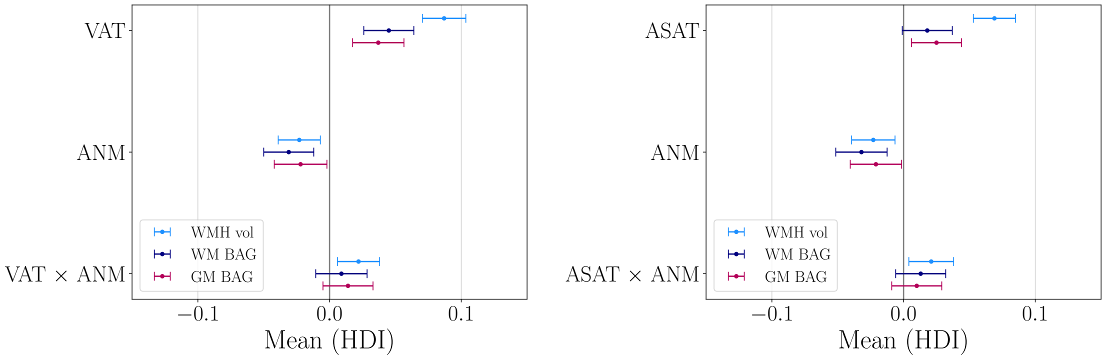

SI Figure 9: Associations between brain measures and visceral adipose tissue (VAT), abdominal subcutaneous adipose tissue (ASAT), age at natural menopause (ANM), and the interaction terms. The points show the means of the posterior distributions for the associations, with error bars indicating the 95% highest density intervals (HDI). GM = grey matter, WM = white matter, BAG = brain age gap, WMH vol = white matter hyperintensity volume.

5.4. Including all ages at menarche/menopause and surgical menopause

When re-running the models without removing outliers for age at menarche and menopause, oophorectomy and/or hysterectomy, the associations and interactions were consistent with the main results as shown in SI Figure 10.

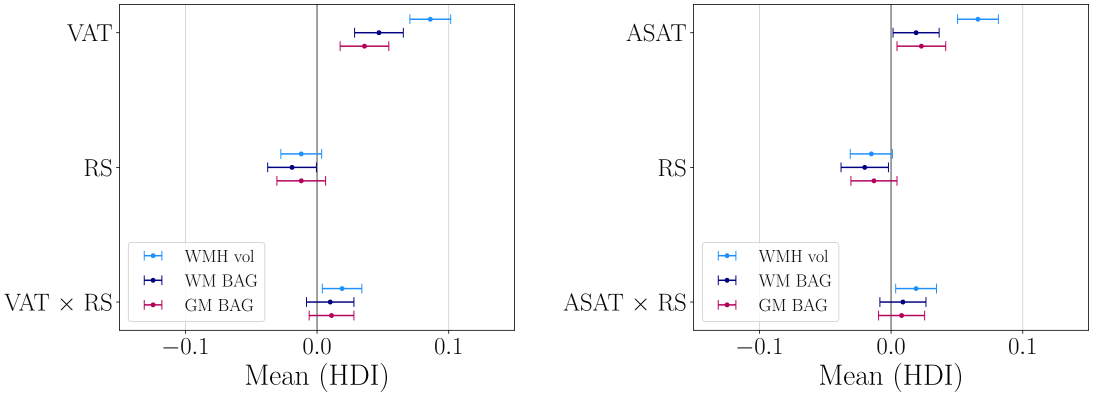

SI Figure 10: Associations between brain measures and visceral adipose tissue (VAT), abdominal subcutaneous adipose tissue (ASAT), and reproductive span (RS) without prior exclusion of outliers for age at menarche and age at menopause, or a history of oophorectomy and/or hysterectomy. The points show the means of the posterior distributions for the associations, with error bars indicating the 95% highest density intervals (HDI). N participants with complete data on all variables = 11,381. GM = grey matter, WM = white matter, BAG = brain age gap, WMH vol = white matter hyperintensity volume.

References

Basser, P. J., Mattiello, J., & LeBihan, D. (1994). MR diffusion tensor spectroscopy and imaging. *Biophysical journal*, *66*, 259–267.

Fieremans, E., Jensen, J. H., & Helpern, J. A. (2011). White matter characterization with diffusional kurtosis imaging. *Neuroimage*, *58*, 177–188.

Fischl, B., Salat, D. H., Busa, E., Albert, M., Dieterich, M., Haselgrove, C., Van Der Kouwe, A., Killiany, R., Kennedy, D., Klaveness, S. et al. (2002). Whole brain segmentation: automated labeling of neuroanatomical structures in the human brain. *Neuron*, *33*, 341–355.

Glasser, M. F., Coalson, T. S., Robinson, E. C., Hacker, C. D., Harwell, J., Yacoub, E., Ugurbil, K., Andersson, J., Beckmann, C. F., Jenkinson, M. et al. (2016). A multi-modal parcellation of human cerebral cortex. *Nature*, *536*, 171.

Jensen, J. H., Helpern, J. A., Ramani, A., Lu, H., & Kaczynski, K. (2005). Diffusional kurtosis imaging: the quantification of non-gaussian water diffusion by means of magnetic resonance imaging. *Magnetic Resonance in Medicine: An Official Journal of the International Society for Magnetic Resonance in Medicine*, *53*, 1432–1440.

Kaden, E., Kelm, N. D., Carson, R. P., Does, M. D., & Alexander, D. C. (2016). Multi-compartment microscopic diffusion imaging. *NeuroImage*, *139*, 346–359.

Kaufmann, T., van der Meer, D., Doan, N. T., Schwarz, E., Lund, M. J., Agartz, I., Alnæs, D., Barch, D. M., Baur-Streubel, R., Bertolino, A. et al. (2019). Common brain disorders are associated with heritable patterns of apparent aging of the brain. *Nature neuroscience*, *22*, 1617–1623.

Voldsbekk, I., Barth, C., Maximov, I. I., Kaufmann, T., Beck, D., Richard, G., Moberget, T., Westlye, L. T., & de Lange, A.-M. G. (2021). A history of previous childbirths is linked to women's white matter brain age in midlife and older age. *Human Brain Mapping*, *42*, 4372–4386.
